# Education for out-of-school children: Unpacking driving factors of vulnerability via digital-learning footprint data in East Africa

**DOI:** 10.23889/ijpds.v8i3.2293

**Published:** 2023-09-11

**Authors:** Bethany Huntington, Nicola Pitchford, James Goulding

**Affiliations:** 1 Department of Psychology, University of Nottingham; 2 N/LAB, NUBS, University of Nottingham

## Introduction & Background

Approximately 617 million children and adolescents worldwide do not possess the foundational skills to live healthy and productive lives. Sub-Saharan Africa is profoundly affected, resulting in social and financial dependency and raising vulnerability to forced marriage, female genital mutilation, and mental health issues. Contextual factors are considered critical in this crisis, yet have received little attention due to a currently insurmountable deficit in traditional census and survey data. Digital footprint data offers a potential route to filling this information gap - particularly in education- and how a learner’s environment can impact digital learning and future well-being.

To address the crisis, the ‘Global Learning’ XPRIZE competition challenged teams to develop software empowering marginalised out-of-school children to learn literacy and numeracy skills. Five finalist teams tested their technology with 2041 children using handheld tablets in 172 remote villages in Tanzania.

## Objectives & Approach

Our study examined factors that can predict improvements in learning outcomes, building on the digital footprint data collected from the children participating in the device intervention in the form of app usage and locational and activity data. Additional geospatial features were engineered based on village coordinates, distance to local amenities, services and transport, variables serving as additional potential indicators of isolation and connectedness. These data were linked with child-level factors, including household composition and literacy levels.

After comparative assessment of machine learning regression models, tree-based models (XGB, RF) were used to establish the optimal predictive performance for literacy and numeracy. Variable importance using SHAP was used to determine which specific contextual variables should be considered before deploying digital interventions to support education and well-being.

## Relevance to Digital Footprints

Utilising digital footprint data to quantify the influence of geospatial and contextual data in digital interventions can offer comprehensive insights to understand and address factors impacting learning outcomes in this context.

## Results

Prior school attendance, home reading environments and high familial literacy were found to be predictors of higher learning outcomes after a technology-based learning intervention. Environments featuring an unemployed caregiver and few siblings were surprisingly consistent positive predictors, suggesting accompanying and focussed caregiver support as valuable for effective development via digital interventions. Proximity to police stations and health centres were revealed as key predictors, indicating the importance of social and physical connectedness in positive learning environments.

## Conclusions & Implications

Targeted improvement of EdTech provision with out-of-school learners promises to help the development of learning outcomes in remote villages and reduce vulnerabilities to correlated social and health issues. However, positive outcomes can only be achieved when appropriate supporting environments surround interventions.

